# Network Diffusion Modeling Explains Longitudinal Tau PET Data

**DOI:** 10.3389/fnins.2020.566876

**Published:** 2020-12-23

**Authors:** Amelie Schäfer, Elizabeth C. Mormino, Ellen Kuhl

**Affiliations:** ^1^Department of Mechanical Engineering, Stanford University, Stanford, CA, United States; ^2^Department of Neurology and Neurological Sciences, Stanford School of Medicine, Stanford, CA, United States

**Keywords:** tau PET, Neuroimaging, model calibration, Alzheimer's disease, network diffusion model

## Abstract

Alzheimer's disease is associated with the cerebral accumulation of neurofibrillary tangles of hyperphosphorylated tau protein. The progressive occurrence of tau aggregates in different brain regions is closely related to neurodegeneration and cognitive impairment. However, our current understanding of tau propagation relies almost exclusively on postmortem histopathology, and the precise propagation dynamics of misfolded tau in the living brain remain poorly understood. Here we combine longitudinal positron emission tomography and dynamic network modeling to test the hypothesis that misfolded tau propagates preferably along neuronal connections. We follow 46 subjects for three or four annual positron emission tomography scans and compare their pathological tau profiles against brain network models of intracellular and extracellular spreading. For each subject, we identify a personalized set of model parameters that characterizes the individual progression of pathological tau. Across all subjects, the mean protein production rate was 0.21 ± 0.15 and the intracellular diffusion coefficient was 0.34 ± 0.43. Our network diffusion model can serve as a tool to detect non-clinical symptoms at an earlier stage and make informed predictions about the timeline of neurodegeneration on an individual personalized basis.

## 1. Introduction

The accumulation of pathological amyloid-β and hyperphosphorylated tau protein is a classical hallmark of Alzheimer's disease that occurs years to decades before a clinical diagnosis is possible (Duyckaerts et al., 2009). The widely accepted amyloid cascade hypothesis is based on the assumption that the abnormal aggregation of amyloid-β is the disease initiator, which then causes a series of pathological events including the production and propagation of misfolded tau protein followed by neurodegeneration, regional atrophy, and ultimately cognitive impairment (Jack and Holtzman, 2013). Even though recent years have brought a better qualitative understanding of the various biomarkers involved in Alzheimer's disease (Jack et al., 2013), little is known about the causal, quantitative, and temporal relationships between those markers. Mathematical models can help establish these relations, but they often lack reliable longitudinal data for model calibration and validation.

Positron emission tomography (PET) is a non-invasive imaging technique that enables the tracking of amyloid and tau distributions in a living brain non-invasively *in vivo* (Johnson et al., 2016; Villemagne et al., 2018). The tau PET tracer [^18^F]-AV-1451 binds to paired helical filaments within tau's neurofibrillary tangles (NFT), as proven in postmortem studies when comparing PET signal to histology (Marquié et al., 2015). Hyperphosphorylated tau plays a central role in disease progression due to its confirmed direct relation to neurodegeneration and cognitive impairment (Bejanin et al., 2017; Xia et al., 2017). This relation was first revealed in postmortem histological analyses showing strong correlations between the location and density of tau neurofibrillary tangles and sites of neurodegeneration (Giannakopoulos et al., 2003). Imaging studies confirmed that the intensity of *in vivo* tau PET signal was strongly correlated to regional tissue atrophy measured in longitudinal magnetic resonance images (MRI) (Gordon et al., 2018; Iaccarino et al., 2018; La Joie et al., 2020).

Today, it has become widely accepted that tau is more closely associated with the neurodegenerative process than amyloid-β (Buckley et al., 2017). The observation of pathological amyloid-β and tau protein is not unique to Alzheimer's disease and is similarly associated with healthy aging (Knopman et al., 2003). However, in Alzheimer's disease patients, the propagation sequence of tau protein differs from the one observed in cognitively unimpaired older adults and seems to follow a consistent, stereotypical and reproducible pattern: In cross-sectional autopsy studies, pathological tau first appeared in the transenthorinal cortex before spreading into neighboring regions in the limbic and temporal cortex. After this, neurofibrillary tangles were found to propagate into a wide range of the association isocortex and finally into the primary sensory cortex (Braak and Braak, 1991; Braak et al., 2006). However, the precise spreading pattern of misfolded tau, from one brain region to another, remains incompletely understood. Evidence from animal models suggests that hyperphosphorylated tau propagates along the brain's anatomical neuronal connections (De Calignon et al., 2012; Liu et al., 2012). This is in line with findings from PET imaging studies, which revealed a striking similarity between patterns of *in vivo* tau PET signal and the brain's connectome (Jones et al., 2017; Pereira et al., 2019). Studies have detected higher PET signal intensity in strongly interconnected regions, indicating increased accumulation of tau in these connectivity hubs (Cope et al., 2018).

Tau PET imaging has only been developed recently and longitudinal studies that follow the spatio-temporal distribution of tau in one and the same subject are still rare. A few longitudinal studies exist, but they are limited to a single follow-up visit (Jack et al., 2018; Harrison et al., 2019). To better understand the spreading of misfolded tau, modeling groups have implemented network diffusion and epidemic spreading models to simulate the propagation of tau through the brain and claim good performance when using functional or structural connectomes as basis for their models (Raj et al., 2012, 2015; Torok et al., 2018; Vogel et al., 2020; Weickenmeier et al., 2019). However, none of these models is validated on longitudinal tau data with multiple points in time. Instead, these studies either base their conclusions on atrophy data by postulating correlations between tau topology and atrophy (Raj et al., 2012, 2015; Torok et al., 2018), or on cross-sectional tau PET images that require additional assumptions regarding the initial conditions and model configuration (Vogel et al., 2020).

Recent studies suggest to model the accumulation and spreading of misfolded protein using partial differential equations on a network model based on the brain connectome (Raj et al., 2012; Iturria-Medina et al., 2014; Henderson et al., 2019). Within this framework, the complex pathogenic cascade of protein production, conversion, aggregation, and clearance is captured in, and simplified to, a Fisher-Kolmogorov model (Fisher, 1937; Kolmogorov et al., 1937; Fornari et al., 2019, 2020). While these models show good qualitative agreement with the pathological stages from histopathology (Braak and Braak, 1991), they have not yet been calibrated and validated with real patient data. A calibrated model of misfolded tau protein would enhance our understanding of disease progression, from a qualitative to a quantitative level. Characterizing the typical time-dependent evolution of disease biomarkers is essential for developing new diagnostic tools to detect non-clinical symptoms at an earlier stage and for evaluating potential new treatments.

Here we use longitudinal tau PET images from 46 subjects to calibrate the parameters of two competing network diffusion models based on either anisotropic intracellular spreading in a connectivity-weighted network or isotropic extracellular spreading in a distance-weighted network. A side-by-side comparison of both models with the longitudinal PET images allows us to test the hypothesis that misfolded tau spreads preferably intracellularly, along neuronal connections. In contrast to previous studies, we do not make artificial assumptions about initial tau seeding or the age at onset. Instead, we directly extract the initial conditions from the first PET scan and use the second, third, and fourth scans for personalized model calibration.

## 2. Materials and Methods

### 2.1. Image Data Selection

Our study uses longitudinal imaging data from the Alzheimer's Disease Neuroimaging Initiative database (ADNI), a multisite, longitudinal, public database of MRI and PET images for normal cognitive aging, mild cognitive impairment, and early Alzheimer's disease ADNI (2020). We include data from 46 participants who have undergone at least three consecutive annual tau PET scans. Of these, 16 are diagnosed as cognitively normal, 9 with significant memory concern, 19 with mild cognitive impairment, and two with clinically confirmed Alzheimer's disease. A total of 26 are classified as amyloid positive based on previously evaluated β-amyloid PET images (Landau et al., 2013). To decrease bias, we conduct our study blind to diagnosis status. All acquired AV1451-PET scans have previously been preprocessed according to standard ADNI protocols (ADNI, 2020) to be co-registered and averaged, and to have a standardized image and voxel size and a uniform image resolution of 8 mm FWHM. For each PET scan, we obtain a corresponding high resolution T1 weighted magnetic resonance image (MRI) from the database, recorded on average within 3 months prior or post PET acquisition. When a concurrent MRI scan is not available, we use data acquired at the closest visit in time. The average time span between longitudinal tau PET scans was 1.0 year, ranging from 0.6 to 2.8 years.

### 2.2. Image Data Analysis

**Figure 1:**
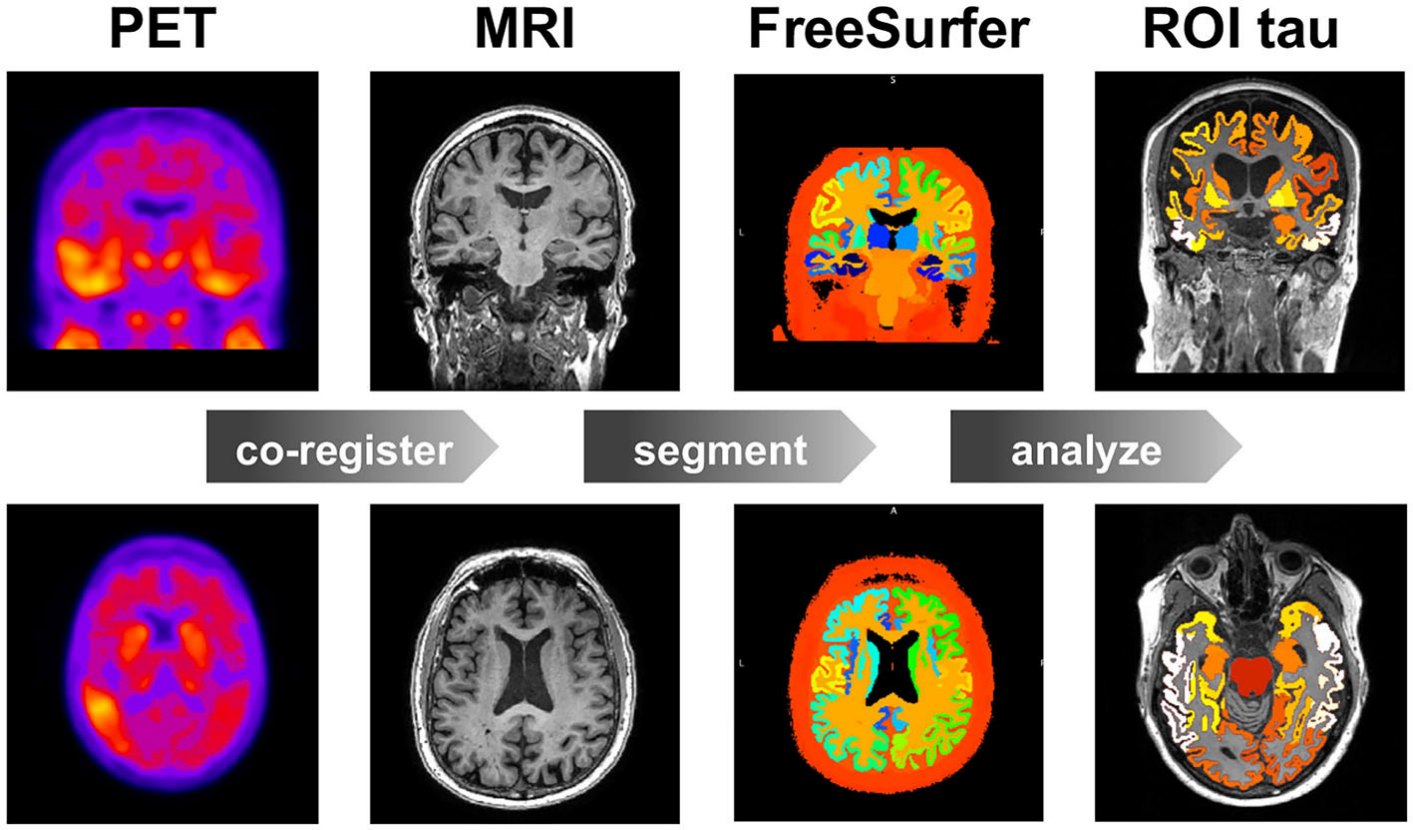
Image data analysis. Workflow for region of interest (ROI) based positron emission tomography (PET) image analysis. For each subject, at each time point, we co-register the PET images to the T1 weighted magnetic resonance images (MRI), which we segment using FreeSurfer to calculate the standardized uptake value ratios (SUVR) for each region of interest (ROI). Our study contains 46 subjects, 3–4 time points, and 83 regions of interest.

For each subject, we analyze the longitudinal PET data using the method summarized in Figure 1 (Baker et al., 2017). Briefly, we co-register the PET images to the corresponding MRI scan using SPM (SPM, 2020) with 4th degree spline interpolation and run a full reconstruction of the T1 MRI using FreeSurfer (FreeSurfer, 2020). This segments the brain into 68 cortical and 45 subcortical regions and allows us to extract regional values of tau binding from the PET images. We define an inferior cerebellar gray matter reference region using the SUIT template (Diedrichsen, 2006), which we reverse normalize into the subject's native T1 MRI space. To create regional standardized uptake value ratios (SUVR), we normalize all regional uptake values with respect to the tracer uptake value from the reference region. Known off-target binding sites, e.g., the basal ganglia and vascular structures like the choroid plexus and dural venous sinuses, have been shown to contaminate the AV1451 PET signal in subcortical regions and the hippocampus (Lowe et al., 2016; Marquié et al., 2017; Lemoine et al., 2018). We exclude these regions from the analysis and focus our model optimization on the 66 remaining cortical regions.

### 2.3. Brain Network Modeling

We model the spreading of hyperphosphorylated tau in the brain as a diffusion process within a network, which we represent as a weighted undirected graph G with *N* nodes and *E* edges. To test the hypothesis of preferred tau spreading along neuronal connections, we create two competing network models, a connectivity-weighted network for anisotropic intracellular spreading and a distance-weighted network for isotropic extracellular spreading.

**Figure 2:**
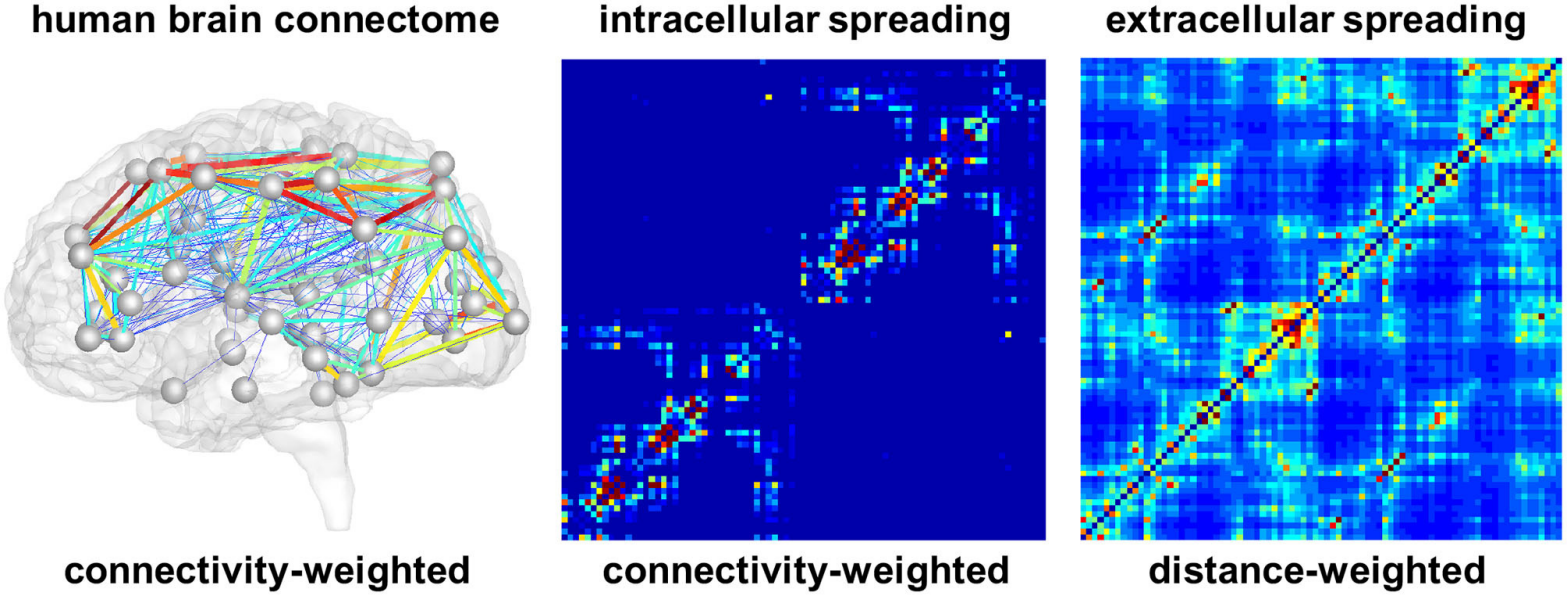
Brain network models. Connectivity-weighted network from the human brain connectome and adjacency matrices of connectivity-weighted intracellular spreading model and distance-weighted extracellular spreading model. The intracellular spreading model features a small number of strong connections within each hemisphere and only few connections between them; the extracellular spreading model features a large number of moderately strong connections across the entire brain. Colors represent the connectivity between two brain regions.

For the connectivity-weighted network, we extract the graph $G^{\text{con}}$ from diffusion tensor MRI data of 418 healthy subjects from the Human Connectome Project (McNab et al., 2013) using the Budapest Reference Connectome v. 3.0 (Szalkai et al., 2017). We map the original graph with *N* = 1, 015 nodes onto a graph with *N* = 83 nodes (Fornari et al., 2019). These 83 nodes correspond to the brain regions extracted in the FreeSurfer segmentation of cortex and subcortex, allowing us to directly compare our model degrees of freedom with the regional tau signal. Figure 2 shows the connectivity-weighted network with strong connections in red and weak connections in blue. In this graph, each edge is weighted by the average number of fibers *n*_*ij*_ detected between two nodes *i* and *j* divided by the average fiber length *l*_*ij*_ along this connection across all 418 brains. This introduces the adjacency matrix of the connectivity-weighted network as $A_{ij}^{\text{con}}=n_{ij}/l_{ij}$. Figure 2 shows the adjacency matrix of the connectivity-weighted intracellular spreading model with a small number of strong connections within each hemisphere and only few connections between them.

For the distance-weighted network, we construct a graph $G^{\text{dist}}$ with the same 83 nodes as the first graph $G^{\text{con}}$. However, for this case, we define an edge between each pair of nodes and weight it by the inverse of the Euclidian distance *d*_*ij*_ between the two nodes. This introduces the adjacency matrix of the distance-weighted network as $A_{ij}^{\text{dist}}=1/d_{ij}$. Figure 2 shows the adjacency matrix of the distance-weighted extracellular spreading model with a large number of moderately strong connections across the entire brain.

### 2.4. Network Diffusion Modeling

Motivated by the hypothesis that tau protein misfolds and spreads in a prion-like fashion (Jucker and Walker, 2011; Fornari et al., 2020), we use a Fisher-Kolmogorov model (Fisher, 1937; Kolmogorov et al., 1937) to characterize the accumulation of pathological tau in the brain (Fornari et al., 2019; Thompson et al., 2020). The model is governed by a single non-linear reaction-diffusion equation that predicts the spatio-temporal evolution of the unknown, the concentration of misfolded protein *c*,

(1) $$\begin{array}{r} \frac{\text{d}c}{\text{d}t}=\nabla \cdot \left(\text{D}\cdot \nabla c\right)+\alpha c\left[1-c\right], \end{array}$$

where **D** and α denote the diffusion tensor and the local production rate of misfolded protein. The production rate α captures the processes of protein production, clearance, and conversion (Fornari et al., 2019). To model diffusion within a network, we discretize Equation (1) on the undirected graphs $G^{\text{con}}$ and $G^{\text{dist}}$. We introduce the concentration of misfolded proteins *c*_*i*_ at all *i* = 1, ..., *N* nodes and express the change in the concentration as

(2) $$\begin{array}{r} \frac{\text{d}c_{i}}{\text{d}t}=-\kappa \overset{N}{\underset{j=1}{\sum }}L_{ij}c_{j}+\alpha c_{i}\left[1-c_{i}\right], \end{array}$$

where κ characterizes the global diffusion between two regions and α the local production or clearance of misfolded protein. A central element of Equation (2) is the weighted graph Laplacian *L*_*ij*_, a square matrix, which we construct from the adjacency matrix *A*_*ij*_. The sum of all elements across each row of the adjacency matrix *A*_*ij*_ defines the degree matrix *D*_*ii*_,

(3) $$\begin{array}{r} D_{ii}=\text{diag}\overset{N}{\underset{j=1,j\neq i}{\sum }}A_{ij}. \end{array}$$

The graph Laplacian *L*_*ij*_, the difference of the degree matrix and the adjacency matrix, summarizes the connectivity of the graph,

(4) $$\begin{array}{r} L_{ij}=D_{ij}-A_{ij}. \end{array}$$

For each subject, we identify a personal diffusion coefficient κ and a personal protein production rate α that best characterize the progression of pathological tau from their individual longitudinal PET scans. Depending on the type of model, we replace the adjacency matrix *A*_*ij*_ in Equations (3) and (4) with the connectivity weighted or distance weighted adjacency matrix, $A_{ij}^{\text{con}}$ or $A_{ij}^{\text{dist}}$. For comparison, we normalize both matrices such that their entries lie within the [0,…,1] interval. Using these normalized matrices, we identify the intracellular or extracellular diffusion coefficient κ and the production rate α.

### 2.5. Parameter Identification

The simulation with the network diffusion model provides a region-specific normalized concentration *c*^sim^ with values between zero, for no misfolded protein, and one, for a maximum misfolded protein concentration, 0 ≤ *c*^sim^ ≤ 1. To map the recorded PET standardized uptake value ratios into a zero-to-one interval, we fit a two-component Gaussian mixture model to the raw PET data from all subjects, time points, and regions. We assume that many regions and subjects are free from pathological tau and use this distribution to identify a tau positivity threshold of 1.1. We set all values below this threshold to zero and map the remaining values *c*^raw^ onto the scaled values *c*^pet^ using the maximum and minimum non-zero PET signals *c*^max^ = max{*c*^raw^} and *c*^min^ = min{*c*^raw^} as *c*^pet^ = [*c*^raw^ − *c*^min^]/[*c*^max^ − *c*^min^], such that 0 ≤ *c*^pet^ ≤ 1. We adopt a least squares optimization to identify the personalized diffusion coefficients κ and production rates α that best reproduce the progression of tau for each subject. Specifically, we optimize the parameter set for the connectivity-weighted and the distance-weighted networks by minimizing the squared error between the simulated concentrations $c_{i,t}^{\text{sim}}$ and the PET recorded concentrations $c_{i,t}^{\text{pet}}$ within one subject for all *i* = 1, ..., *n*_roi_ regions of interest and all *t* = 1, ..., *n*_visit_ follow-up visits,

(5) $$\begin{array}{r} \text{e}\text{r}\text{r}=\overset{n_{\text{roi}}}{\underset{i=1}{\sum }}\overset{n_{\text{visit}}}{\underset{t=1}{\sum }}\beta {\left[c_{i,t}^{\text{sim}}\left(\kappa ,\alpha \right)-c_{i,t}^{\text{pet}}\right]}^{2}. \end{array}$$

Here, β is a scalar factor to improve numerical stability, *n*_roi_ is the number of cortical regions for which we have high confidence data according to section 2.2.

### 2.6. Model Performance

For comparison, we perform the optimization on three null models to probe the importance of the different model components. For the first null model, we leave out the term for local protein production, α = 0, and optimize solely the diffusion coefficient κ. For the second null model, we leave out the diffusion term, κ = 0, and optimize solely the protein production rate α. For the third null model, we assume that tau is neither spreading nor produced, κ = 0, α = 0, which implies that the protein concentration in each region remains constant across all follow-up visits. We identify the subjects with positive production rate, α > 0. We assume these are the subjects with pathological tau expression who are more likely to develop or have signs of Alzheimer's disease and focus our further analysis on this subgroup. For the two network models and the three null models, we compare the performance in terms of the global residual error across all subjects, all cortical regions of interest, and all follow-up visits. We plot the observed vs. predicted values and calculate a correlation coefficient to illustrate the quality of the respective fits. We use paired-sample t-tests to determine whether differences in subject-wise prediction error between different models are significant. Furthermore, we use Fisher's R-to-z transform to determine whether differences in correlation coefficients between different models are significant.

### 2.7. Model Prediction

Our dataset only spans a time period of 2–3 years whereas the accumulation of tau typically spans a period of around 15 years (Bateman et al., 2012). We use the connectivity-weighted intracellular model and distance-weighted extracellular model to predict the tau concentrations across the brains of all 46 subjects for a time window of 15 years. This allows us to explore the long-term performance of the two models, compare their predictions against histopathological findings, and test our hypothesis of intracellular spreading.

## 3. Results

### 3.1. Regional Tau PET Concentration

**Figure 3:**
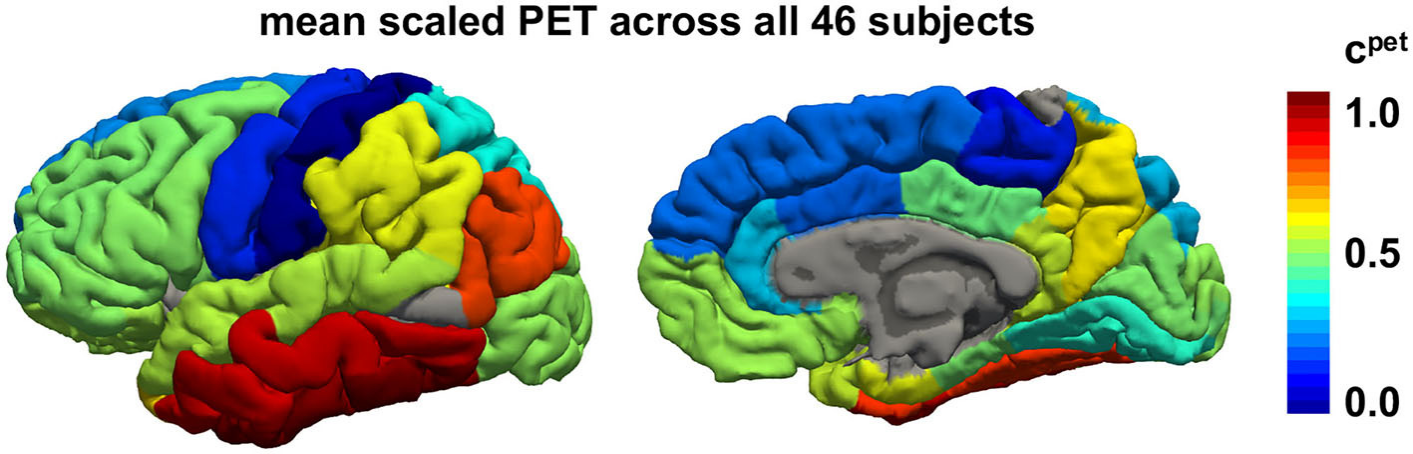
Regional tau PET concentration. Mean tau concentration from PET scans across all 46 subjects with 3–4 annual scans across all brain regions. Red regions consistently exhibit high tau loads in all subjects while blue regions tend to be free of tau in most subjects.

Figure 3 illustrates the regional average standardized uptake value ratios across all subjects and visits on a template brain surface. The temporal lobes show the highest tau PET signal intensity, followed by occipital and frontal lobes. The precentral and postcentral gyrus display the lowest tau signal intensities.

### 3.2. Longitudinal Tau PET Concentration

**Figure 4:**
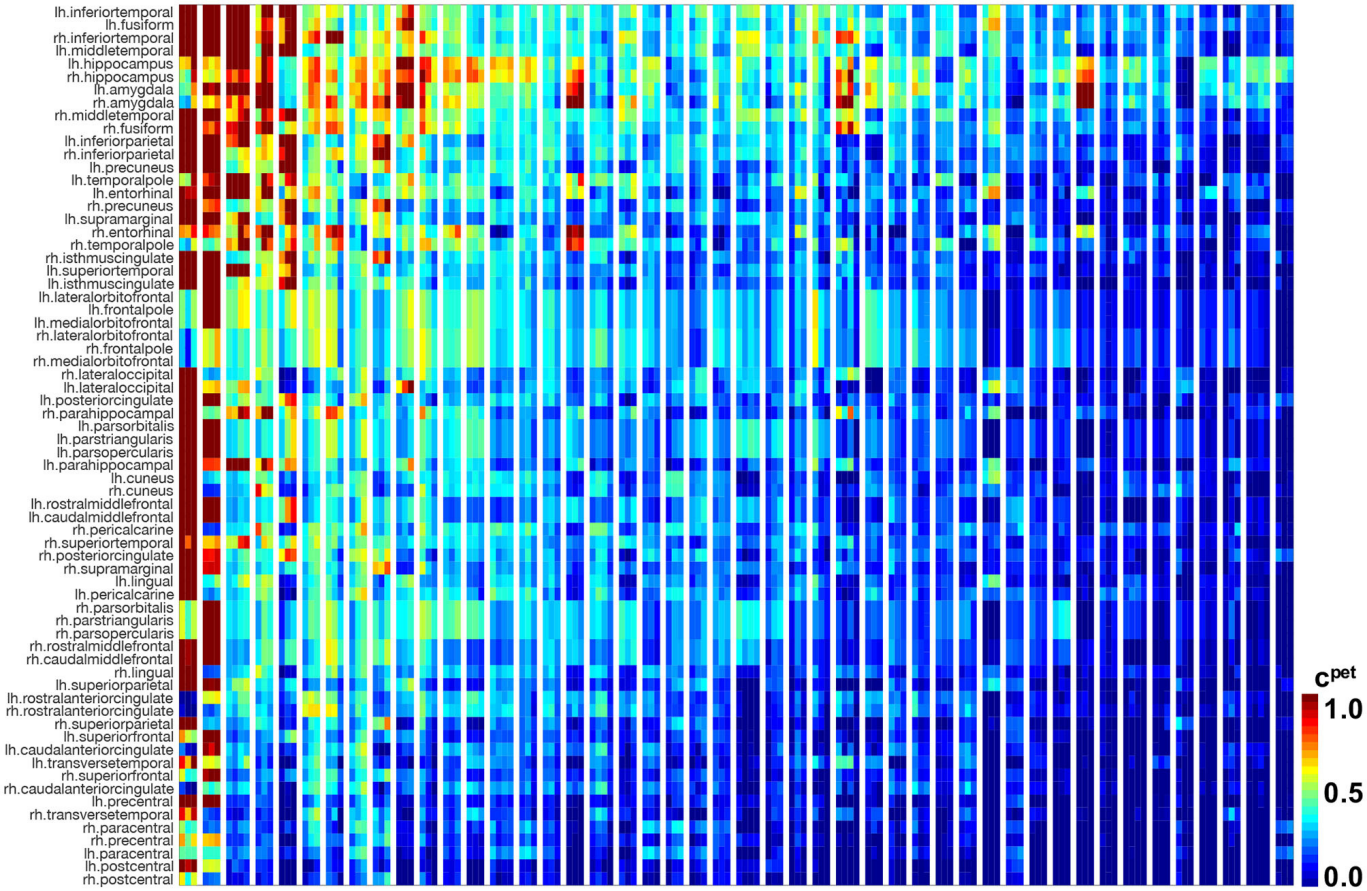
Longitudinal tau PET concentration. Standardized uptake value ratios from PET scans for 46 subjects with 3–4 annual scans in 66 cortical regions and the hippocampus. Regions on the vertical axis are sorted by mean tau load, from top to bottom. Subjects on the horizontal axis are sorted by mean tau load across all regions and visits, from left to right. Each block of columns represents data for one subject. Within each block, each subcolumn represents data from one annual PET scan.

Figure 4 illustrates the results of our image analysis for all 46 subjects, shown as blocks of columns, all time points, shown as columns, and 66 cortical regions as well as the hippocampus, shown as rows. The color code indicates the normalized tau standardized uptake value ratios. On the horizontal axis, subjects are ordered according to their overall tau load averaged across all regions and visits, with the most affected subject on the left and the least affected subject on the right. On the vertical axis, regions are ordered with respect to their overall tau load averaged across all subjects and visits with the regions showing the highest involvement at the top and regions with the lowest involvement at the bottom. The inferiortemporal, middletemporal, and fusiform gyrus, the amygdalae, and the hippocampus are the regions that are most consistently affected with high tau signals. They are followed by the inferiorparietal lobule, the precuneus, the entorhinal cortex, and the temporalpole. Interestingly, we see bands of moderately but consistently affected regions involving the orbitofrontal cortex, the frontalpole, and the inferiorfrontal gyrus including the parsorbitalis, parstriangularis, and parsopercularis. For most regions, the right hemisphere seems to be less affected by tau than the left hemisphere. This asymmetry is especially prominent for the temporalpole, the inferiorfrontal gyrus, the middlefrontal gyri, and the posterior cingulate cortex. The precentral, paracentral, and postcentral gyrus are the least affected regions. The hippocampus and amygdalae appear to be affected above average in most subjects, even in subjects with very low tau signal in all other regions of interest.

### 3.3. Parameter Identification

**Figure 5:**
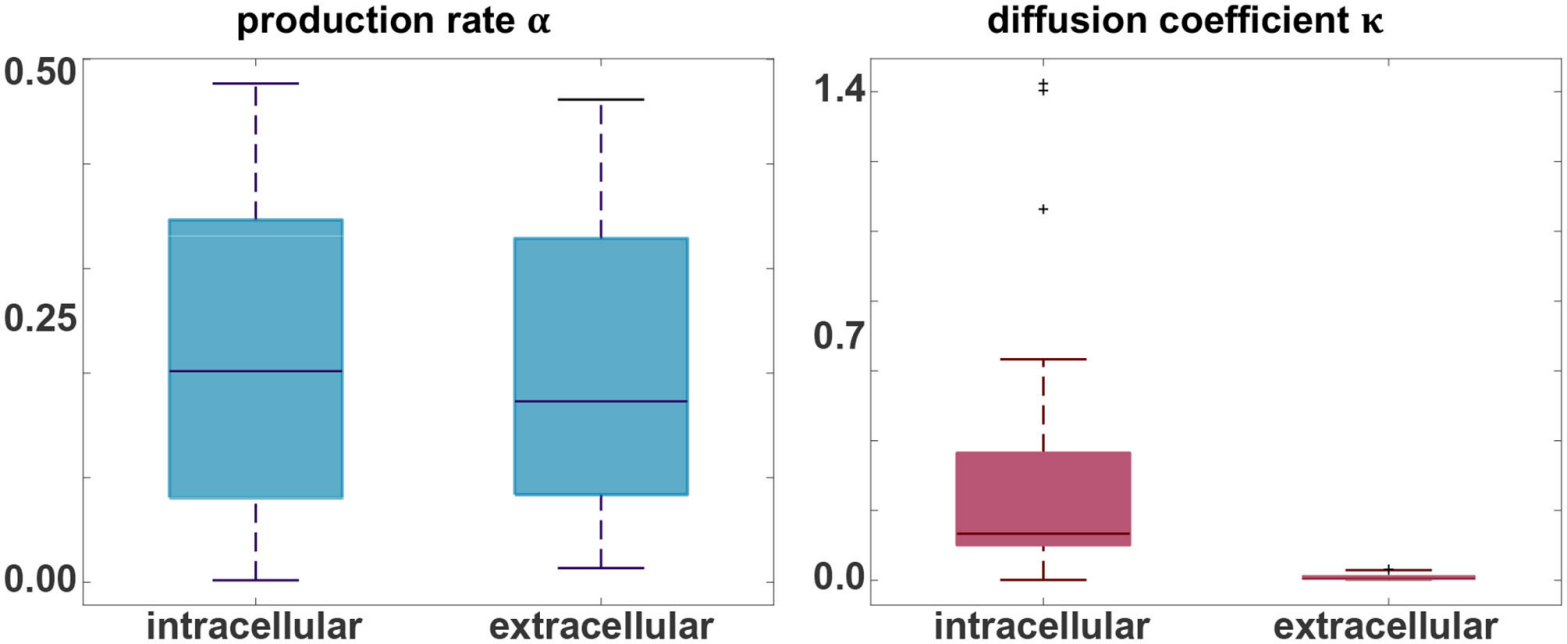
Parameter identification. Personalized production rate α and diffusion coefficient κ for the intracellular and extracellular diffusion models, only including the 21 subjects with a positive production rate. For the connectivity-weighted intracellular spreading model, α = 0.21 ± 0.15 and κ = 0.34 ± 0.43. For the distance-weighted extracellular spreading model, α = 0.20 ± 0.14 and κ = 0.01 ± 0.01.

Figure 5 indicates the ranges of the personalized production rates α and diffusion coefficients κ for the connectivity-weighted intracellular and distance-weighted extracellular diffusion models for 21 subjects. Out of the 46 subjects, 21 exhibited a longitudinal tau signal that was best fit using a positive protein production rate, α > 0 and 25 exhibited a signal best fit using a negative production rate, α < 0. We postulate that the 21 subjects with a positive production rate are the subjects with pathological tau expression who are more likely to develop Alzheimer's disease and focus on the results of this subgroup. The majority of these 21 subjects, 16 out of 21, were identified with a positive amyloid status. Of the remaining five, two had no amyloid status reported, one reported a positive cerebrospinal fluid amyloid status, and two reported a negative PET and cerebrospinal fluid amyloid status. While the production rates for the connectivity-weighted intracellular and distance-weighted extracellular models with α = 0.21 ± 0.15 and α = 0.20 ± 0.14 are in a similar range, the diffusion coefficient for the connectivity-based model with κ = 0.34 ± 0.43 is notably larger than for the distance-weighted model with κ = 0.01 ± 0.01. This difference in the diffusion coefficients compensates the difference in magnitude of the entries in the adjacency matrices of the two models, which we can see in Figure 2. For the connectivity-weighted intracellular model, the diffusion coefficient κ shows three outliers associated with subjects that exhibit more and faster spreading than the average subject.

### 3.4. Model Performance

**Figure 6:**
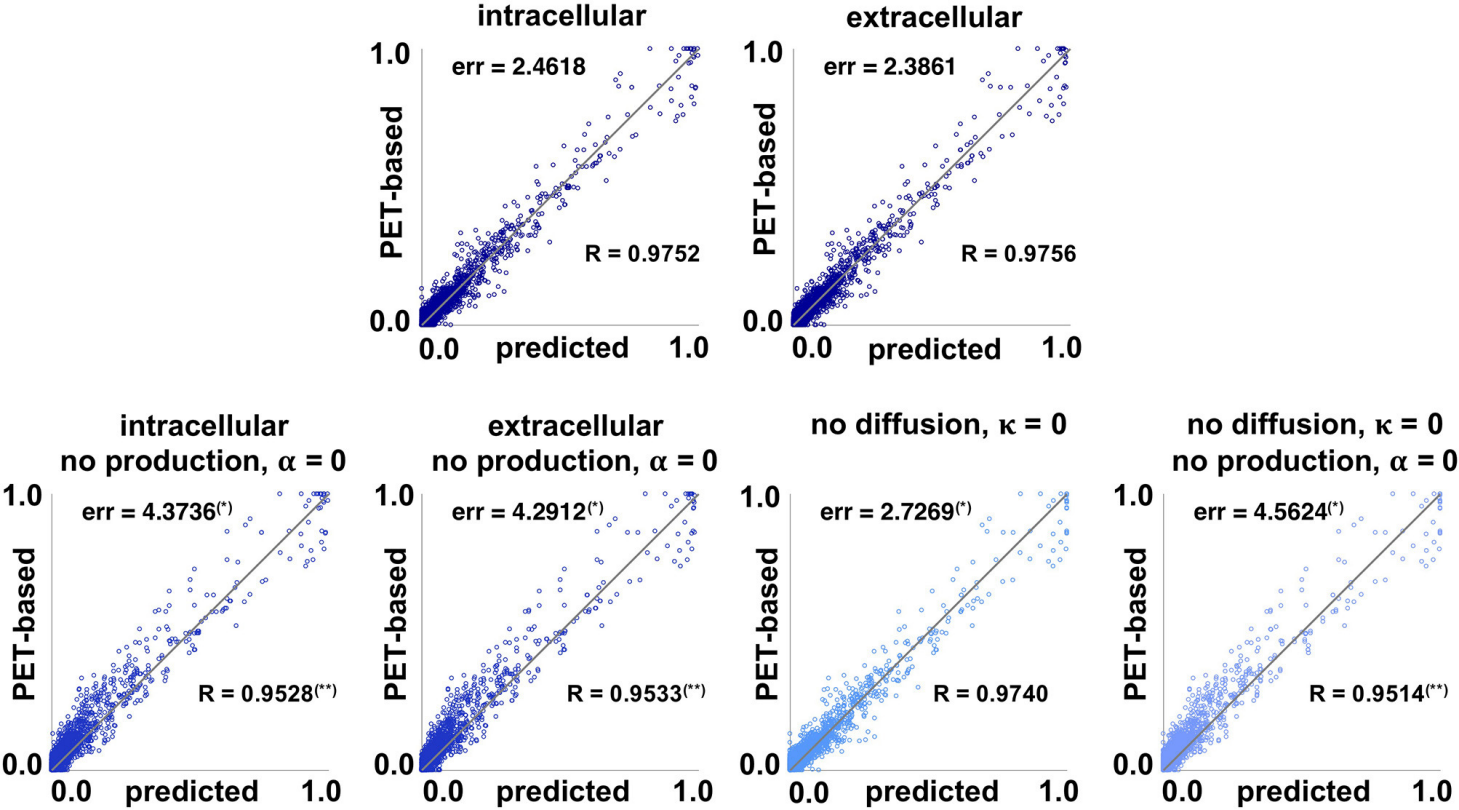
Model performance. Simulated concentration *c*^sim^ and PET-based concentration *c*^pet^ of pathogenic tau protein for intracellular and extracellular network diffusion models and null models without production, without diffusion, and without both. Each data point represents the simulated and PET-based concentration for one subject, one visit, and one region of interest. The further a data point is away from the gray line, the worse the prediction. The global residual error err of each model measures the overall prediction error of each model. (*) indicates a subject-wise error significantly higher than for the full model in paired-sample t-test. The correlation coefficient R measures the correlation strength between prediction and observation for each model. (**) indicates a correlation coefficient significantly lower than for the full model using Fisher's R-to-z transform.

Figure 6 summarizes the performance of the two network models compared to the four null models described in section 2.6, the intracellular and extracellular spreading models without production, the pure production model without diffusion, and a model without diffusion and production. Each data point represents the simulated concentration *c*^sim^ and PET-based concentration *c*^pet^ for one subject, one visit, and one region of interest. For an ideal fit, all points would lie on the gray diagonal line.

The lowest residual error, and best correlation between the simulated and PET-based concentration was achieved with the distance-weighted extracellular model with err = 2.3861 and R = 0.9756, followed closely by the connectivity-weighted intracellular model with err = 2.4618 and R = 0.9752. A paired-sample t-test showed no significant difference between the subject-wise errors associated with extracellular and intracellular models (p_err_ = 0.07). Fisher's R-to-z transform showed no significant difference between the correlation coefficients associated with extracellular and intracellular models (p_R_ = 0.59). Eliminating the production term from the diffusion equation, α = 0, significantly increased the prediction errors for both the intracellular and extracellular models, to err = 4.3736 (p_err_ = 5.7*e*−04) and err = 4.2912 (p_err_ = 6.0*e*−04). The correlation coefficients significantly decreased to R = 0.9528 (p_R_ = 0.0) and R = 0.9533 (p_R_ = 0.0) when eliminating the production term. The prediction error of the null model without diffusion, κ = 0, with err = 2.7269 is significantly higher than with the full models (p_err_ = 0.002, p_err_ = 0.0015), but significantly lower than with the null models without production (p_err_ = 0.0012, p_*err*_ = 0.0016). This is not surprising, when considering how close to zero the diffusion coefficient was for the distance-weighted extracellular model in Figure 5. Notably, the correlation coefficient R = 0.9740 is not significantly lower for the model without diffusion compared to the full models with diffusion (p_R_ = 0.0012). The final null model, which assumes that all tau concentrations remain constant at the value from the first scan, results in the largest residual error of err = 4.5624 with significantly higher subject-wise prediction errors than all other null models (p_err_ ≤ 0.0051) and significantly lower correlation strength R = 0.9514 (p_R_ = 0.0). On the personalized level, the distance-weighted extracellular model performs better for 13 subjects and the connectivity-weighted intracellular model performs better for the remaining 8. The model performance suggests that the production term α is a critical component of the tau pathology model that significantly affects the quality of model prediction. Additionally, we see that the data imply existing tau propagation from region to region, even though the diffusion term seems to have overall less importance than the production term. Finally, even with the most simplified null model for which the tau PET concentration does not change in time, the data points are, even though slightly scattered, still relatively close to the diagonal line that marks the perfect correlation between simulation and PET data. This emphasizes the limitation of the current approach, which only contains longitudinal data from 2 to 3 years. We will continuously update our model as more time points become available to address this limitation.

**Figure 7:**
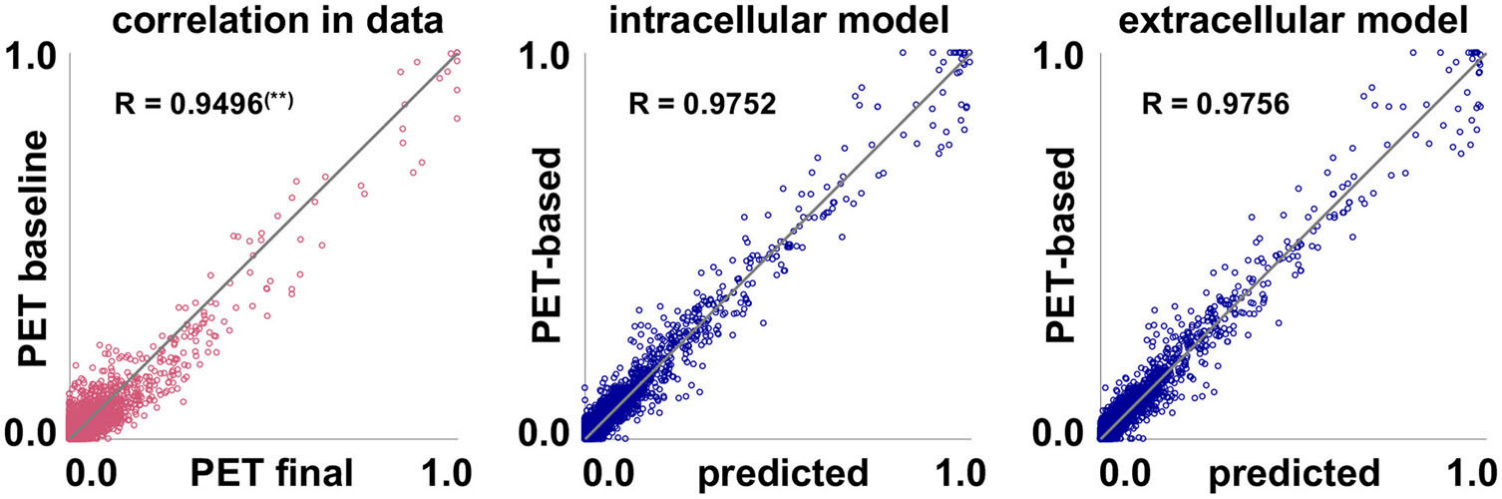
Model performance. Inherent data correlation. Baseline and final PET-based concentrations *c*^pet^, and simulated concentration *c*^sim^ over PET-based concentration *c*^pet^ of pathogenic tau protein for intracellular and extracellular network diffusion models. Each data point represents the PET-based concentration for one subject, one region of interest, and one visit. (**) Correlation coefficient R is significantly lower than for the two proposed models.

Figure 7 illustrates the correlation between baseline and final observed PET data for all subjects and regions of interest. The plot shows that the data inherently exhibits a high correlation, with a correlation coefficient of R = 0.9496. This again highlights how small the observed changes in tau load are over the observation period of 2–4 years. Fisher's R-to-z transform however confirms that the correlation significantly increases with our proposed intracellular and extracellular models (p_R_ = 0.0).

### 3.5. Model Prediction

To investigate the predictive nature of the connectivity-based intracellular and distance-based extracellular models, we simulate the spatio-temporal pathogenic tau distribution for all 21 subjects with positive production rate throughout a period of 15 years using the models from sections 2.3, 2.4 with the personalized initial conditions, production rate α, and diffusion coefficient κ.

**Figure 8:**
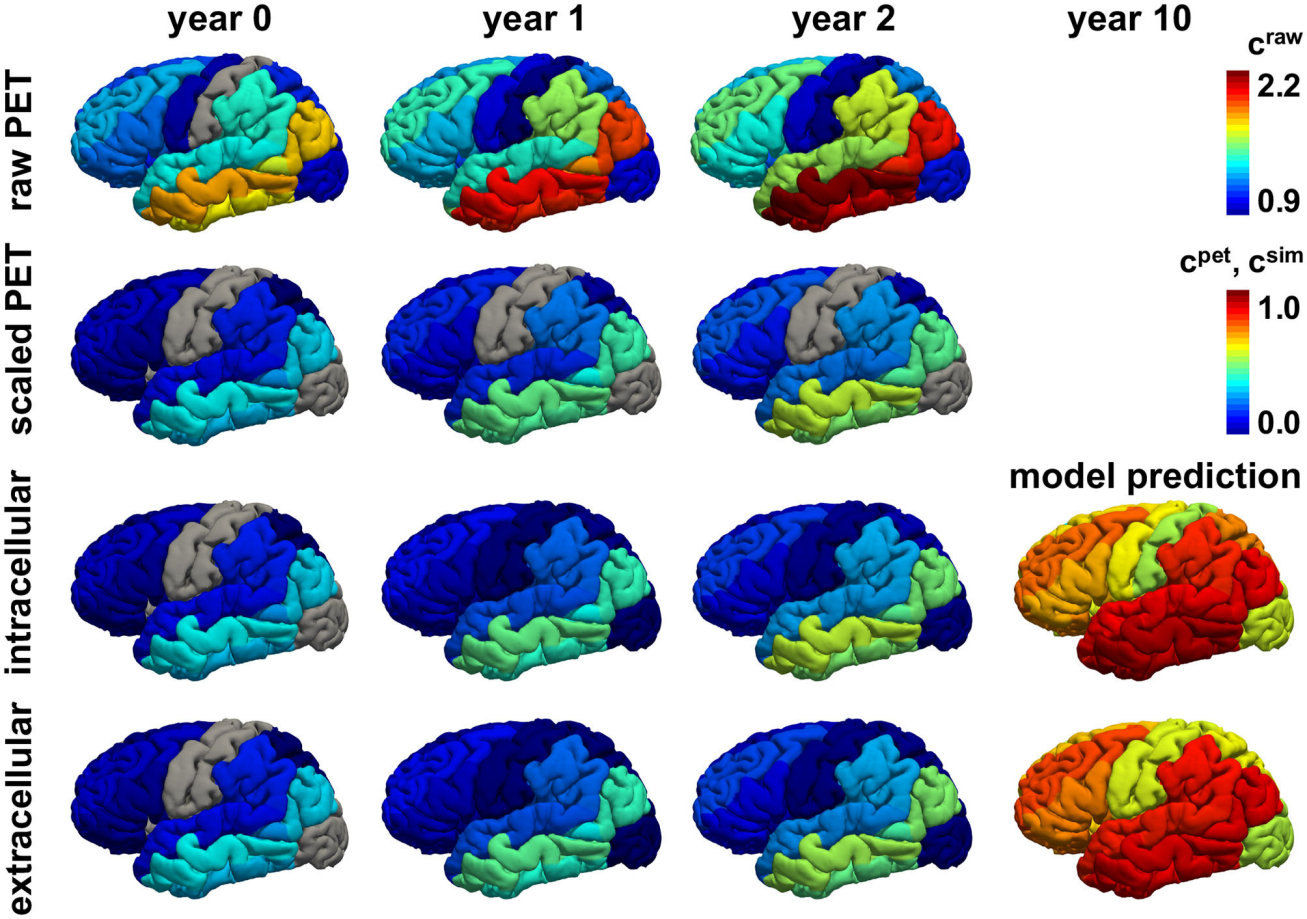
Personalized model prediction. Regional tau concentrations from raw and scaled standardized uptake value ratios *c*^raw^ and *c*^pet^ vs. simulated tau concentrations *c*^sim^ with a connectivity-weighted intracellular and a distance-weighted extracellular model for personalized initial conditions, production rates, and diffusion coefficients of subject #12 from Figures 9, 10. Lateral view, left hemisphere.

**Figure 9:**
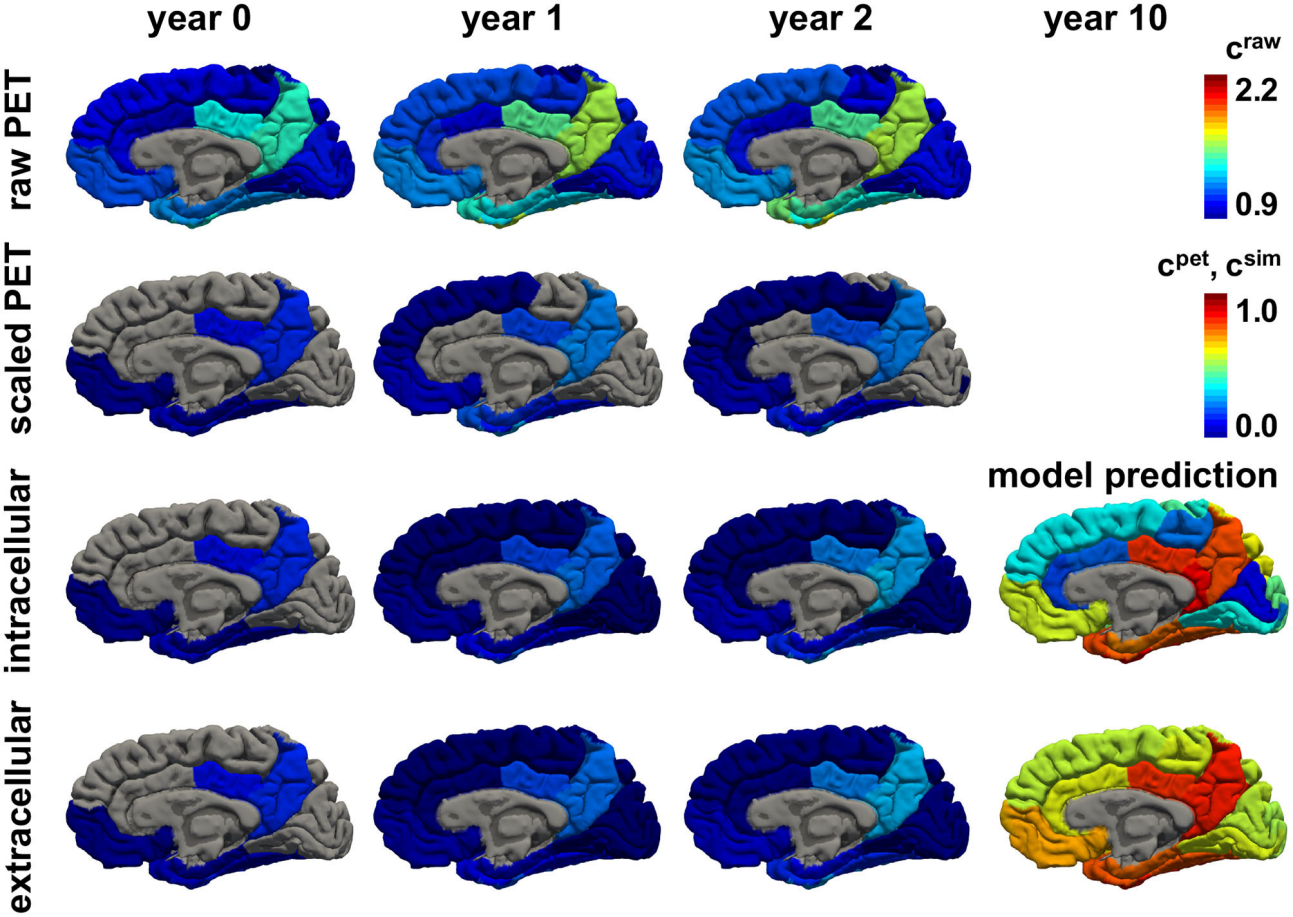
Personalized model prediction. Regional tau concentrations from raw and scaled standardized uptake value ratios *c*^raw^ and *c*^pet^ vs. simulated tau concentrations *c*^sim^ with a connectivity-weighted intracellular and a distance-weighted extracellular model for personalized initial conditions, production rates, and diffusion coefficients of subject #12 from Figures 9, 10. Medial view, right hemisphere.

Figures 8, 9 show the personalized model predictions for a single subject, with personalized initial conditions, production rates, and diffusion coefficients. The first and second row showcase the PET tau concentrations from the raw and scaled standardized uptake value ratios *c*^raw^ and *c*^pet^ for 3 years. The third and fourth row show the simulated tau concentrations *c*^sim^ from the connectivity-weighted intracellular model with α = 0.422 and κ = 0.133 and the distance-weighted extracellular model with α = 0.437 and κ = 0.007 for the first 3 years and for year 10. Both models first follow the observed PET concentration closely with only marginal differences in the predictions. However, after 10 years, the predicted tau concentration pattern from the intracellular model is much more heterogeneous than the concentration from the extracellular model. This is especially visible in the medial view of the right hemisphere in Figure 9, where the colors of the intracellular model still range from dark blue to red, whereas in the extracellular model predicts values in the color range from yellow to red.

**Figure 10:**
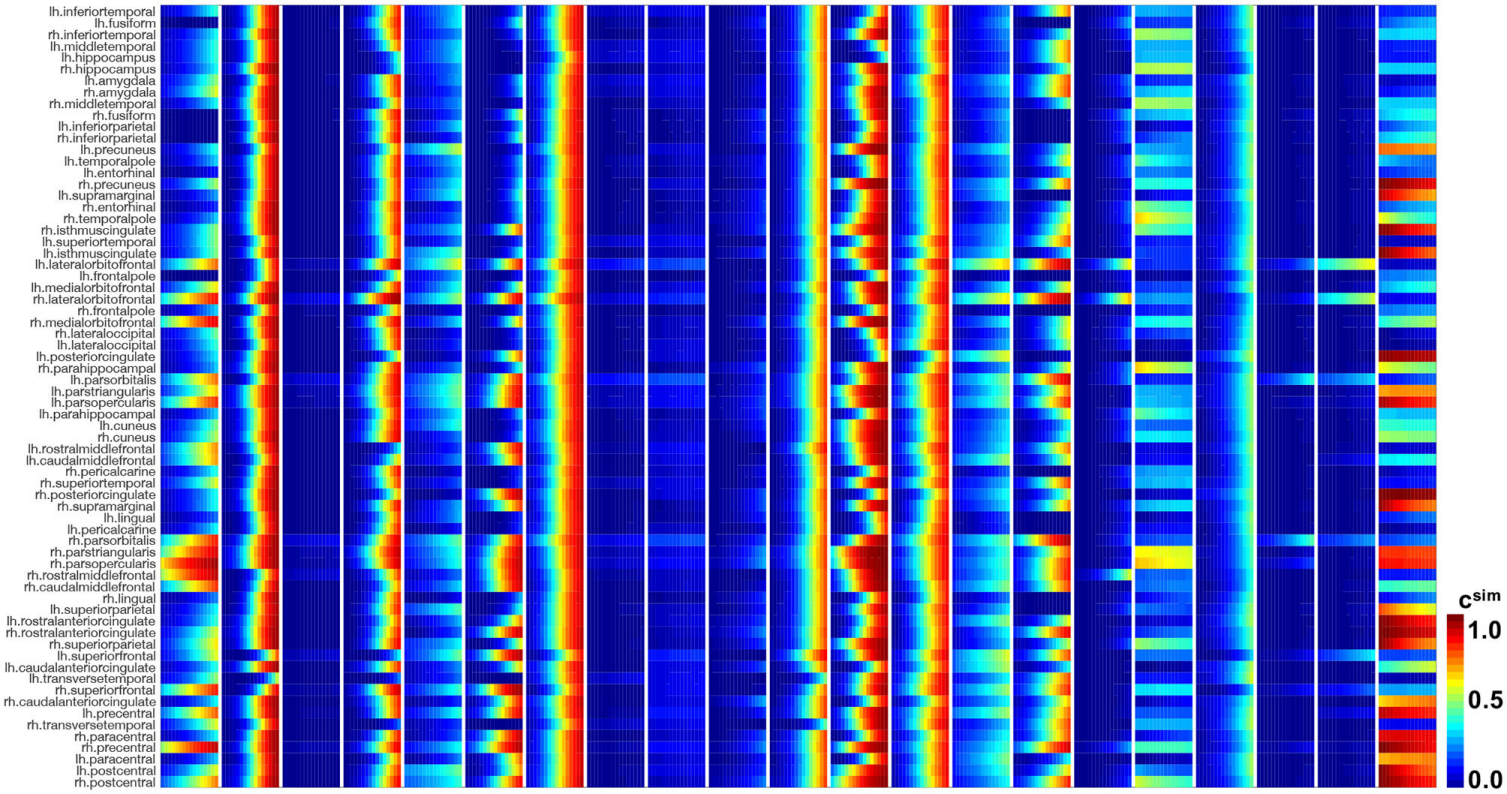
Model prediction of intracellular model. Simulated tau concentrations *c*^sim^ with connectivity-weighted intracellular model for 21 subjects for 15 years in 66 cortical regions and the hippocampus. Each block of columns represents the simulation for one subject with their personalized initial conditions, production rate α, and diffusion coefficient κ. Within each block, each subcolumn represents simulated concentrations for 1 year.

**Figure 11:**
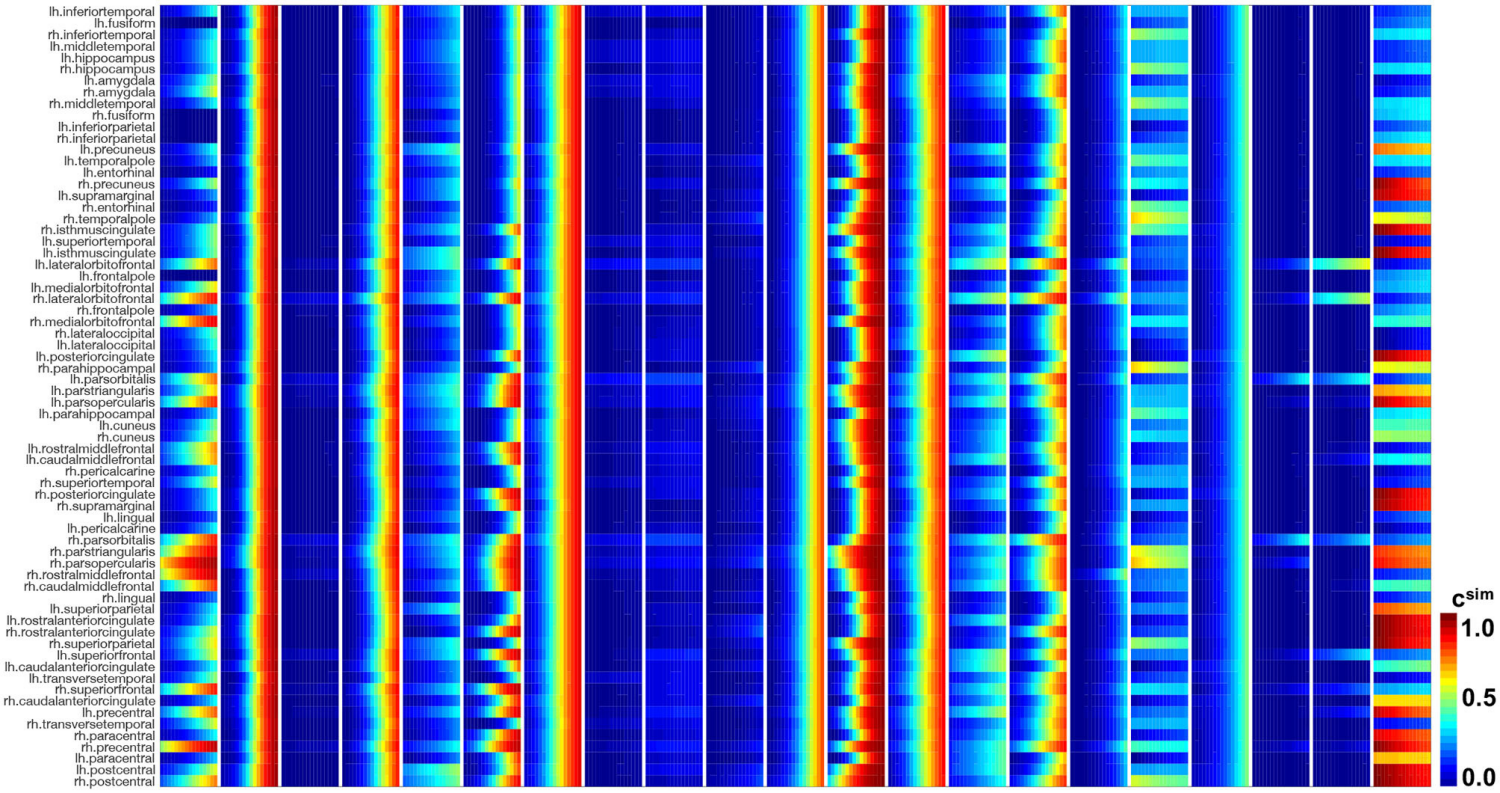
Model prediction of extracellular model. Simulated tau concentrations *c*^sim^ with connectivity-weighted intracellular model for 21 subjects for 15 years in 66 cortical regions and the hippocampus. Each block of columns represents the simulation for one subject with their personalized initial conditions, production rate α, and diffusion coefficient κ. Within each block, each subcolumn represents simulated concentrations for 1 year.

Figures 10, 11 show the predictions for the connectivity-weighted intracellular model and the distance-weighted extracellular model. Each block of columns represents the simulation for one subject for the 15-year window. For some subjects, the predicted long-term response is similar for both models. However, in most subjects, the predicted pathological pattern differs between the intracellular and extracellular approach. Interestingly, the intracellular model maintains a staggered and sequential involvement of different regions within one subject, the extracellular model predicts a more homogeneous and smoothened spatial distribution of pathological tau protein. In this sense, the intracellular model preserves the inhomogeneous topology of the tau spreading process, in which individual regions of the cortex begin to express high concentrations of pathological tau in a sequential way, with successively more regions affected over time. In contrast, the extracellular model predicts a gradual increase in pathological tau protein, but involves all regions homogeneously at the same time.

## 4. Discussion

The objective of this study was to identify personalized parameters of a network diffusion model for pathogenic tau propagation using longitudinal tau PET data. As part of this study, we tested the hypothesis that misfolded tau spreads through the brain primarily along neuronal connections. To test this hypothesis, we compared the performance of two competing network models, a connectivity-based intracellular spreading model and a distance-based extracellular spreading model. Ultimately, both modeling approaches resulted in good correlations between the observed tau PET concentrations and the simulated tau concentrations. While we were not able to confidently confirm our hypothesis about the transport mechanism of tau because of the limited amount of available longitudinal data, the long-term predictions of tau pathology support our intracellular spreading hypothesis. The intracellular spreading model predicted a more heterogeneous tau spreading that agrees better with the well-accepted histopathological staging than the extracellular spreading model.

Previous studies that model the propagation of tau pathology have used cross-sectional PET (Vogel et al., 2020) or atrophy data (Raj et al., 2012; Torok et al., 2018) for mode validation. However, even though longitudinal tau pathology has successfully been correlated with atrophy patterns (La Joie et al., 2020), there are multiple factors calling into question the use of tissue atrophy as a direct predictor for tau pathology. First, there is a considerable time lag between tau accumulation and neurodegenerative tissue atrophy in Alzheimer's disease (Bejanin et al., 2017; Harrison et al., 2019), the exact magnitude of which is unknown. Second, tau accumulation is not a unique cause for atrophy during aging and disease, so directly inferring tau topology from atrophy measurements could be misleading. Opting for tau PET data allows for a more direct quantitative validation. However, using cross-sectional PET data for calibration of a time-dependent model introduces a certain bias, as it requires additional assumptions for initial conditions at disease onset, age at onset and propagation speed. Cross-sectional studies also neglect inter-individual differences in disease progression. Here, instead, we evaluate the performance of tau pathology models calibrated with longitudinal tau PET data. This inherently removes the need to make assumptions about initial conditions and minimizes bias.

From the longitudinal data, we inferred a sequence of regions earliest and most affected by misfolded tau. This sequence is—despite some slight differences in the initial regions—in line with the results from cross-sectional studies (Cho et al., 2016; Vogel et al., 2020) and with the well-accepted histopathological staging (Braak and Braak, 1991; Braak et al., 2006): Histologically, neurofibrillary tangles were first observed in the transentorhinal cortex before spreading into proper entorhinal cortex and the hippocampus. The amygdala is one of the regions affected next, followed by a more widespread range of regions in the inferior facies of the temporal and occipital lobe and finally other regions of the isocortex in temporal, frontal, occipital and parietal lobe. The only regions found to be relatively spared of pathology even in late disease stages were the primary sensory areas and primary motor field in the precentral and postcentral gyrus. Our data roughly follows this sequence, with the hippocampus and amygdalae affected early on and in many subjects and the postcentral and precentral gyri affected the least across all subjects. The results of our analysis imply less involvement of the entorhinal cortex than expected according to Braak's stages. This discrepancy could originate from technical limitations of our PET image analysis. The entorhinal cortex is a very small structure and the standardized PET image resolution is low due to the multi-site nature of the ADNI database. Thus, uptake value measurements in the entorhinal region could be compromised through bleed in from other regions and tissues.

Unavoidably, all segmentation and co-registration algorithms are associated with a certain error. For FreeSurfer's parcellation and segmentation algorithm, the entorhinal cortex is associated with a relatively low correlation between manual and automated segmentation when compared to other regions (Desikan et al., 2006; McCarthy et al., 2015). Therefore, errors in segmentation, co-registration and the low PET resolution may have caused inaccuracies in our measurements for the entorhinal cortex and led to the resulting low rankings of 15 and 18 in our sequence. However, a longitudinal PET study has recently shown that early tau accumulation can be more widespread and is not necessarily confined just to the entorhinal cortex in all individuals (Jack et al., 2018). The tau PET signal in the hippocampus is known to be often compromised by off-target binding to the nearby choroid plexus (Lemoine et al., 2018). This could explain, why we observe consistently high binding to the hippocampus in our dataset, even in subjects that have a very low tau load in all other regions. Overall, the results from our longitudinal image analysis are reasonable, especially when considering the integral image quality, and are in general agreement with existing literature.

We identified the parameters of two network diffusion models using longitudinal PET data of 46 subjects. We then focused on the 21 subjects with a positive protein production rate. We postulate that those individuals are most likely to follow the typical Alzheimer's disease cascade with prion-like tau pathology. The majority of those subjects had been classified as amyloid positive, which supports our hypothesis, and indicates an abnormal accumulation of amyloid-β prior to the observed accumulation of tau. The distribution of personalized model parameters from the model optimization process exhibits a notable variance considering inter-individual differences in disease progression. In a recent study, which compared the performance of a connectivity-based to a distance-based network model with respect to cross-sectional tau PET data of 312 subjects, the connectivity-based model was clearly superior in reproducing the data (Vogel et al., 2020). However, when directly comparing the two models with respect to our longitudinal tau data, we did not see a clear superiority of the connectivity-weighted model. In fact, both models performed nearly equally well, resulting in good correlations between simulated and observed tau PET distributions over time. This is likely due to the limited time span of our data, covering disease development only within 3–4 years. We found the change in tau burden—especially the propagation from region to region—to be so low that even a model without diffusion term would simulate the data acceptably well. Solely based on our parameter identification, it is thus not possible to solidly confirm the hypothesis that tau spreads along the brain's connectome. However, when comparing the long-term prediction of the two models, we found that the connectivity-weighted intracellular model predicts more defined and distinct distributions of tau that are in line with the histopathologically observed heterogeneity of tangle spread. As more longitudinal tau PET data become available over the course of the next years, we will revisit our analysis to draw more sophisticated conclusions and confirm or disprove our hypothesis.

While our connectivity-based network diffusion model is able to describe the spatio-temporal evolution in our data well, it is still associated with some residual error. These shortcomings of the model may arise from the fact that there could be other factors influencing the spread of misfolded tau through the brain. For example, it has been suggested that differences in gene expression between regions could cause regionally varying production and clearance rates of healthy or misfolded tau and thereby affect the progression of pathology (Grothe et al., 2018). This regional vulnerability could be included in our model in the future by allowing the production rate α to be a region-specific parameter informed by gene expression.

We have previously proposed and examined a coupled non-linear finite element model for the simulation of Alzheimer's disease related atrophy dependent on local tau pathology (Weickenmeier et al., 2018; Schäfer et al., 2019). The model parameters of our network diffusion model could directly be applied to inform neurodegeneration models. Since more and more studies are confirming a qualitative correlation between tau pathology and regional brain atrophy measurements (Harrison et al., 2019; La Joie et al., 2020), our next step will be to characterize this correlation more quantitatively using our coupled model informed by the here presented longitudinal tau PET data on the one hand and longitudinal atrophy measurements from structural MRI of the same patients on the other hand.

This study comes with several limitations, some of which can naturally be addressed as more data become available: First, the size of our cohort was limited to a small number of subjects with a sufficient number of follow-up tau PET scans. Our data show that there is a lot of inter-subject variability in tau PET data. Including more subjects, will increase statistical power and make it easier to deduce clear trends for disease progression and typical tau pathology. To counteract potential overfitting, in the future, we will use Bayesian hierarchical modeling, a statistical approach allowing for the inference of personalized parameters drawn from a common distribution. This will allow us to account for commonalities between all subjects while simultaneously attesting to inter-subject variability. Second, the maximum number of visits per subject was limited to four. As subjects will return for future scans, we will be able to follow the observations in individual subjects over longer periods of time and evaluate the longitudinal performance of our model. While histopathology shows that truncated tau proteins prevail and the presence of hyperphosphorylated tau decreases as the disease advances, our study does not show a clear trend in this direction, which could be a result of the limited amount of data and the short time window of observation. We will keep adding future studies of years 5 and 6 to our analysis and hope to see a clearer trend in the future. Third, since ADNI is a multi-center study, the images are acquired on various scanner types with various different resolutions. To balance these differences in image quality, all data used in this work were standardized to the lowest common resolution. This low resolution in PET images intensifies partial volume effects, since multiple tissue types can be contained in one voxel, resulting in contamination of regional intensities through wash-out and bleed-in.

## 5. Conclusion

We proposed a new method to calibrate different network diffusion models using longitudinal tau PET data. We identified personalized model parameters that characterize the individual nature of tau pathology progression in 46 subjects. Specifically, we used the misfolded protein production rate to stratify all subjects into those with a positive production rate, more likely to develop neurodegeneration, and those with a negative production rate. For the subjects with a positive production rate, we found a mean production rate of 0.21 ± 0.15 and a mean intracellular diffusion coefficient of 0.34 ± 0.43. Our results suggest that the propagation of misfolded tau from region to region is slow in most subjects—barely measurable within a time frame of 3 to 4 years—calling for further investigation once more longitudinal data become available. Our overall findings support the hypothesis that tau pathology propagates across the brain along structural neuronal connections. Ultimately, our method allows us to quantitatively characterize personalized tau pathologies in their spatio-temporal characteristics, which can in turn be used to inform models of other related disease biomarkers, including regional atrophy.

## Data Availability Statement

Publicly available datasets were analyzed in this study. This data can be found here: Alzheimer's Disease Neuroimaging Initiative: adni.loni.usc.edu.

## Author Contributions

AS was responsible for conception and design of the study, data analysis and interpretation, and draft of the manuscript. EM and EK contributed to and guided study conception and design and provided critical revision of the manuscript for intellectual content. All authors approved the final version of the article to be published.

## Conflict of Interest

The authors declare that the research was conducted in the absence of any commercial or financial relationships that could be construed as a potential conflict of interest.
